# Supplementary figures and images for: SGLT2 Inhibition by Intraperitoneal Dapagliflozin Mitigates Peritoneal Fibrosis and Ultrafiltration Failure in a Mouse Model of Chronic Peritoneal Exposure to High-Glucose Dialysate
Source: Biomolecules. 2020 Nov 19;10(11):1573. doi: 10.3390/biom10111573 (PMC7699342; doi:10.3390/biom10111573)

## Supplementary Figure 1

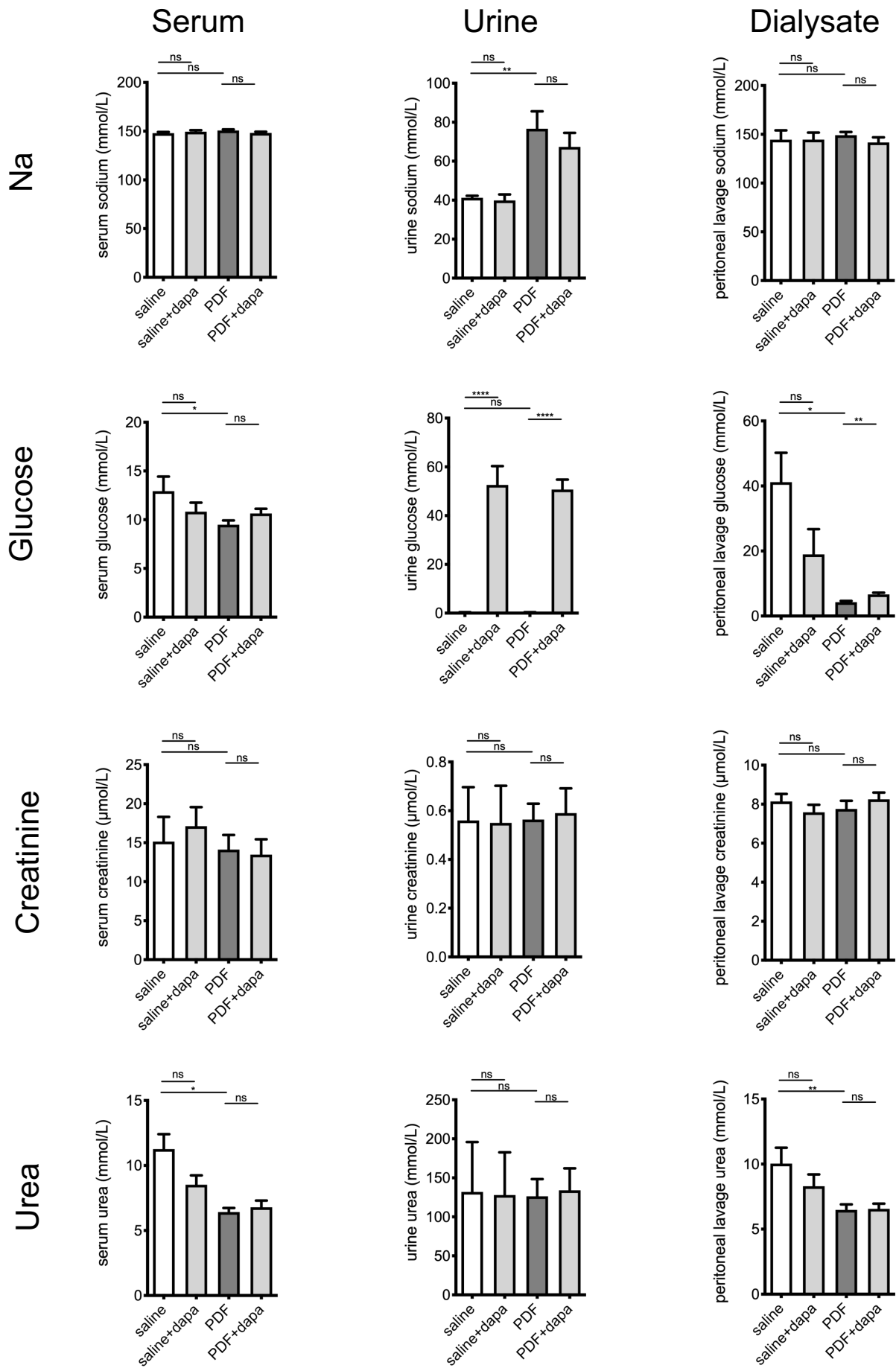

## Supplementary Figure 2

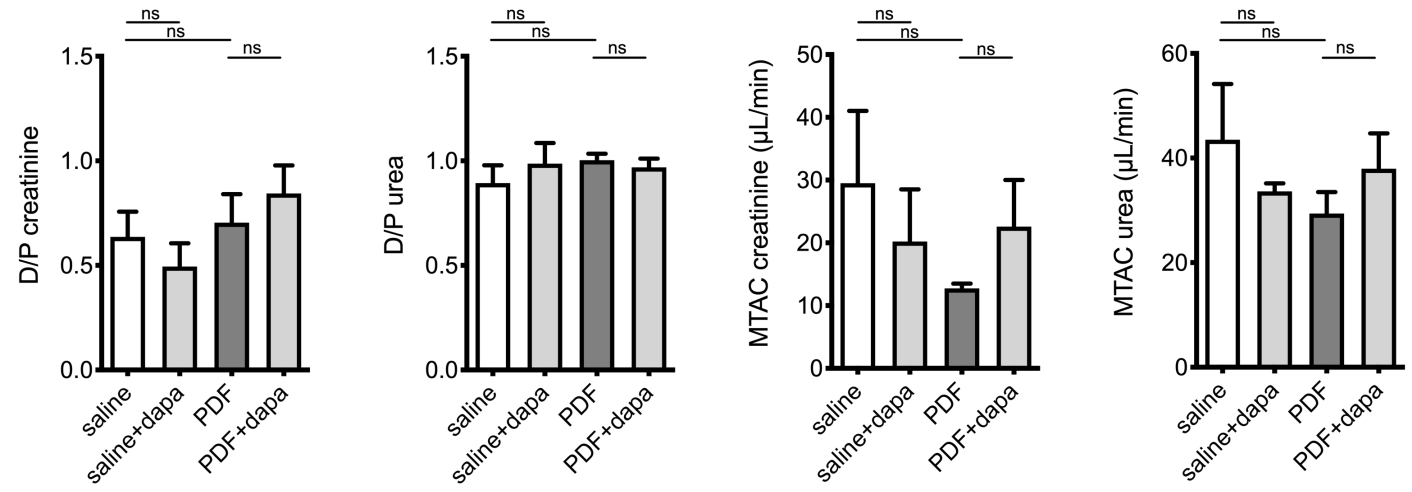

Supplement: Supplementary file 1 [file biomolecules-10-01573-s001.pdf]
